# Assessing the efficacy and safety of Craniosacral therapy for migraine: A single center randomized controlled trial

**DOI:** 10.1097/MD.0000000000035431

**Published:** 2023-11-10

**Authors:** Guangya Jiang, Saichao Ma, Jinfeng Zhao, Ming Zhang, Yan Li, Wenli Chen, Lin Cui, Liuyun Jia

**Affiliations:** a Department of Neurology, Yellow River Central Hospital of Yellow River Conservancy Commission, Zhengzhou, China; b Department of Rehabilitation, Yellow River Central Hospital of Yellow River Conservancy Commission, Zhengzhou, China; c The Department of Gerontology, Zhengzhou University Province People’s Hospital, Zhengzhou, China; d Department of Health Management Center, Yellow River Central Hospital of Yellow River Conservancy Commission, Zhengzhou, China; e Department of Neurological Intensive Medicine, Yellow River Central Hospital of Yellow River Conservancy Commission, Zhengzhou, China.

**Keywords:** complementary light-touch sham treatments, Craniosacral therapy, headache frequency, Headache Impact Test-6, migraine

## Abstract

**Objective:**

Design a feasible study to assess the efficacy and safety of Craniosacral therapy (CST) in the treatment of migraine, using a rigorous and innovative randomized controlled study design involving complementary light-touch sham treatments (CLST) as an attention control intervention.

**Methods:**

This was a single-center, randomized, cross-over placebo-controlled experimental design. A total of 87 participants who suffered migraine attacks from 4 to 9 per month were randomly assigned into either 2 weekly units of CST or CLST for 4 weeks. And then the 2 groups were crossed and continued treatment for 4 weeks plus a follow-up observation for 4 weeks. As the primary outcome measures, Headache Impact Test-6 (HIT-6) and headache frequency were assessed every 4 weeks (at baseline, week 4, week 8 and week 12). The secondary outcome was the scores of Headache Disability inventory (HDI) and the Hamilton Anxiety Scale (HAMA) as well as the adverse events.

**Results:**

All 87 individuals had been screened for eligibility, of which 60 were licensed for the study. The difference of HIT-6 and headache frequency between the 2 groups was not significant at the baseline. But the headache frequency and HIT-6 of 2 groups were all declined respectively after the CST at week 4 (group A) and week 8 (group B) than before (*P*^☆^= 0.01 < 0.05, 95% CI, −3.06 to −1.87; *P*^※^= 0.01 < 0.05, 95% CI, −3.52 to −2.53; *P*^1A^ = 0.01 < 0.05, 95% CI, 4.55–11.7; *P*^2B^ = 0.01 < 0.05, 95% CI, −11.78 to −6.01) while the changes were not obvious after CLST with previous treatment. The scores and frequency of fourth evaluation showed that there was no significant increase or decrease in both the 2 groups. Besides, we found that the mean scores of HIT-6 for all participants, compared with the baseline, were decreased significantly after the 3 round treatments (*P*^3A^ = 0.01 < 0.05, 95% CI, −13.12 to −6.4; *P*^3B^ = 0.01 < 0.05, CI, −12.73 to −6.69). We also showed the similar result in the scores of HDI and HAMA.

**Conclusion:**

The results indicated that standardized CST was both effective and safe in alleviating the migraine intensity and frequency as well as the headache-related disability. Further larger research is needed.

## 1. Introduction

Migraine is a common disabling condition that spans the globe.^[1]^ According to the review by Burch et al, migraine affects approximately 1 out of every 6 American adult population and 1 in 5 women over the past 3-month period.^[2]^ Unlike other chronic diseases, people who are usually healthy, young and meddle-aged are more likely to get sick and women are more prone than men, especially for those aged 18 to 44 years.^[3]^ Although migraine itself does not reduce life expectancy and the morbidity decreases as people age, it can have considerable impact on peoples’ lives and present a significant socioeconomic burden.^[4]^ Those who are experiencing severe migraine may be tired to do daily activities or even absent from work and become bedridden.^[5]^ In addition, repeated migraine attacks can lead to anxiety, depression, cognitive decline, injury of vascular endothelium, and even increase the risk of stroke, which may be related to the pathogenesis of migraine.^[6–8]^

According to the definition of International Headache Society, migraine is a recurrent headache disorder manifesting in attacks lasting 4 to 72 hours (when untreated or unsuccessfully treat).^[9]^ The pathogenesis of headache may be due to disturbance in the brain, where both nerve impulses and chemicals play a part.^[9]^ The typical characteristics of the headache are unilateral location, pulsating quality, moderate or severe intensity, aggravation by routine physical activity (e.g., walking or climbing stairs) and association with nausea, vomiting and/or photophobia and phonophobia.^[9]^

Migraine affects people quality of life and work ability and social activities. Symptoms of migraine are still difficult to control, despite the modification of diet, sleep, aerobic exercise and relaxation as well as the availability of medications are both used for clinical treatment. Furthermore, overuse of medication for the treatment of frequent episodic migraine is a risk factor for developing chronic daily headache.^[10]^ And many patients wish to avoid medication because of the side effects, contraindications, comorbidities or other reasons. The research and evaluation of non-pharmacological or alternative interventions of migraine is therefore warranted. Available evidence suggests that traditional Chinese medicine including acupuncture, massage, yoga, biofeedback, and meditation have a positive effect on migraine and tension headaches.^[11]^ In a review of complementary therapies for chronic pain by Haller, Craniosacral therapy (CST) is a typically treatment for agony of the back and neck, headache and associated stress-related and mental health problem.^[12]^ This mate-analysis also conclude that CST can reduce the use of conventional pain medications and improve daily functioning in a variety of conditions.^[12]^ It is a popular non pharmacological method that uses standardized applications of mild fascia traction, release, and relaxation techniques based on individual palpation limitations, releasing limitations and adjusting the skull and spine until pelvic balance, for the treatment or prevention of neck pain, migraine, and neuromuscular dysfunction. So, we design a feasible study to assess the efficacy and safety of CST in the treatment of migraine, using a rigorous and innovative randomized controlled study design involving complementary light-touch sham treatments (CLST) as an attention control intervention.

## 2. Materials and methods

### 2.1. Design

**Figure 1.**
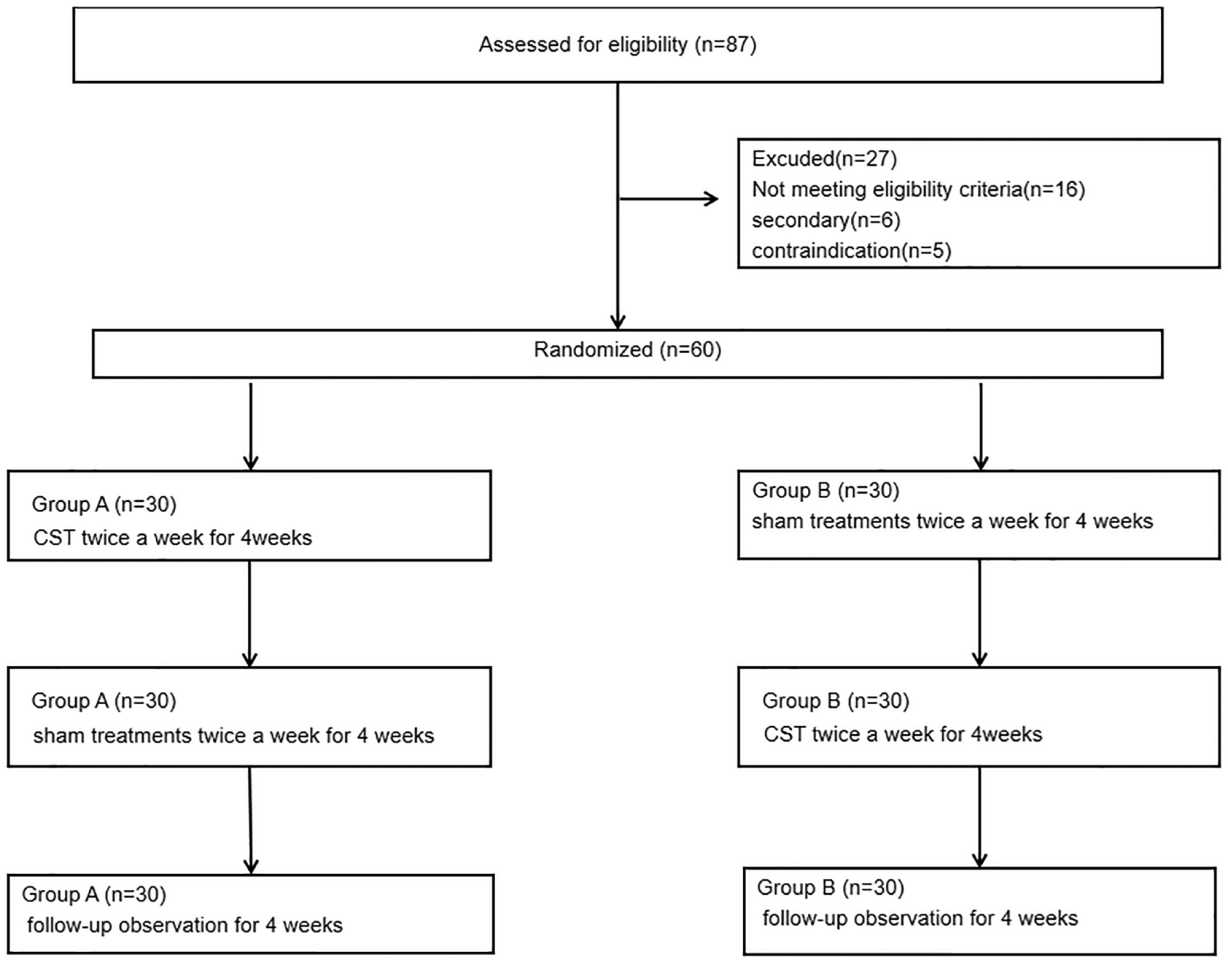
Research process.

This was a single-center, randomized, cross-over, placebo-controlled trial design, completed by neurologists and trained professional rehabilitation therapist. We design a feasibility study to assess the efficacy and safety of CST in the treatment of migraine, using a rigorous and innovative randomized controlled study design involving CLST as an attention control intervention. Figure 1 displays the overall design and subject flow during the study period. After the baseline assessment, participants were equally divided into group A and group B. Headache Impact Test-6 (HIT-6) and frequency of migraine as well as Headache Disability inventory (HDI) and Hamilton Anxiety Scale (HAMA) were collected every 4 weeks: at baseline before treatment sessions, week 4 and week 8 after either CST or CLST intervention and week 12 (4 weeks follow up). Before patients recruitment, the trial protocol had been approved by the ethics committee of Yellow River Central Hospital of Yellow River Conservancy Commission (Protocol NO: 2020-13). All participants signed informed consent before enrollment. The assessment was conducted in the form of questionnaires and questionnaires, and the whole treatment process was completed with the participation of neurologists and Physiotherapist. CST is a soft, noninvasive manual treatment. Theoretically, it is a safe operation and can be applied to a wide range of people. If there is no contraindication, there is generally no special adverse event. However, we still use questionnaires and self-reports to collect adverse events. For example, whether there are symptoms such as worsening headaches, dizziness, fatigue, neck pain, numbness in the limbs, etc. As a subject, you have the following responsibilities: to provide true information about your medical history and current physical condition; Inform the research doctor of any discomfort they have experienced during this study period; Tell the research doctor if they have recently participated in other studies or are currently participating in other studies. You can choose not to participate in this study, or notify the researcher at any time to withdraw from the study. Your data will not be included in the research results, and any medical treatment and rights will not be affected as a result. If you require other treatment, or if you do not comply with the study plan, or if there is a study related injury or any other reason, the study physician may terminate your participation in this study. The study was conducted from 2020.04-2021.04 at Yellow River Central Hospital of Yellow River Conservancy Commission.

### 2.2. Participants

The neurologist used guidelines from the international classification of headache disorders third edition to select patients from hospital where he works and invited them to participate in the study.^[9]^ If they accepted, the first author contacted them and introduced the study to each of them. Table 1 displays Eligibility of Subjects for Clinical effectiveness Trial of CST. The participants should follow the conventional treatment were developed by neurologists, including modification of diet and sleep, aerobic exercise and relaxation as well as the medications for acute treatment of migraine. They were not allowed to use any alternative form of treatment during the study time. Subjects can notify the researcher at any time to withdraw from the study, and your data will not be included in the study results, and any medical treatment and rights will not be affected as a result.

**Table 1:** Eligibility of subjects for clinical effectiveness trial of Craniosacral therapy.

Inclusion criteria	Exclusion criteria
Subjects aged 18–60 yr	Serious depression, anxiety or psychosis
Either gender	Taking psychiatric medication within the previous 3 mo
Meet ICHD 3^rd^ edition criteria for migraine	Major medical illness under treatment
Headache frequency/month: 5–9	Pregnancy
Headache history >2 yr	Clotting disorders
Accept treatment protocol	Headaches caused by other diseases
Able to attend 2 weekly treatments	Head or neck injury in the past 2 yr
Able to be evaluated once every 4 wk	History of skull, neck, and spine surgery
	Cardiac Pacemaker
	Head or neck implanted device (e.g., artery stent)

International Classification of Headache Disorders, defined by expert members of the International Headache Society.

### 2.3. Criteria for inclusion and exclusion

Criteria for inclusion (Table 1) and conventional medicine treatment were developed by neurologists according to the international classification of headache disorders third edition.^[9]^ Participants aged 18 to 60 years, either gender, were included if they had a diagnosis of migraine with or without aura at least for 2 years and the frequency from 4 to 9 per month. The criteria of headache frequency was that patients with more than 5 headache per month could be considered as suffering from failure of conventional therapies. The exclusion criteria include not meeting the eligibility as well as secondary headaches and contraindications.

### 2.4. Randomization and blinding

First, after signed the concealed allocation protocol, the subjects were randomly divided into 2 groups to receive different non-pharmacological treatments. We adopted simple randomization and used the number table grouping method for grouping: group A for CST and group B for CLST. Instead, they were told that 2 different CST techniques would be tested. After 4 weeks, group A and group B were swapped. Second, throughout the study period, the investigator assessing the outcome remained unaware of the allocation of subjects. Third, the statistician who analyzed the results was also blinded to the group assignment.

### 2.5. Intervention

We chose 3 rehabilitation therapists who had received unified craniosacral treatment training for craniosacral treatment and 3 other rehabilitation therapists who also had received unified other treatment training for sham treatment. This research design period lasted 12 weeks, and every 4 week was 1 round of treatment. CST or CLST twice a week, 1 hour each time. HIT-6 and headache frequency were assessed at baseline and right after every treatment round. In the first round, groups A received conventional medicine plus CST and group B received conventional medicine plus CLST. And then interchanged between Group A and Group B in the second round of treatments. Neither group received treatment in the third round, just waiting for the fourth evaluation.

#### 2.5.1. Craniosacral therapy.

Since the brain, spinal cord, cranial muscle fascia and all related structures are the content of the craniosacral system, its restrictions or imbalance will directly affect any or all aspects of the performance of the central nervous system.^[13]^ Therefore, CST was designed to release restrictions and adjust the balance of the cranium and the spine up to the pelvis using standardized application of gentle fascial traction, release, and unwinding techniques in accordance with the respective palpated restrictions. First, the participants laid on their backs and the body completely relaxed. The therapist conducted an assessment of the craniosacral system, and then took notes on the assessment and treated the connective tissue restrictions of the body, neck, all of the cranial bones and underlying tissues. The therapist put 2 thumbs behind the patient ears, overlapped the palms, wrapped the patient neck, and slowly and regularly adjusted the frequency of the cerebrospinal fluid flow. The techniques applied included frontal and parietal lift, medial compression of the parietal bones, release of the sagittal suture and the atlanto-occipital joint, compression-decompression of the sphenobasilar and the temporomandibular joints, cranial base release, release of the hyoid diaphragm and the thoracic inlet, dural tube traction, respiratory and pelvic diaphragm release, lumbosacral and sacroiliac decompression, fascial unwinding of the neck/shoulders.^[14]^ The operator gently touched the affected finger through small adjustments such as relaxation and tension, so as to relieve the patient too fast or too slow craniosacral rhythm, and restored it to a calm and gentle flow state. During the treatments, tension over the pelvis was also relieved, as was in the sutured bones of the skull and the facial bones.

#### 2.5.2. Complementary light-touch sham treatments.

CLST was applied on standardized anatomic areas, equal to those treated with CST. The CLST was also completed by 3 therapists who had received unified training. The CLST protocol was designed to mimic the length of the CST protocol in the treatment session, the sequence of interactions with the therapist, and the terms of the overall treatment experience.

### 2.6. Outcome measures and study instruments

#### 2.6.1. Primary outcome: HIT-6 and Headache frequency.

**Figure 2.**
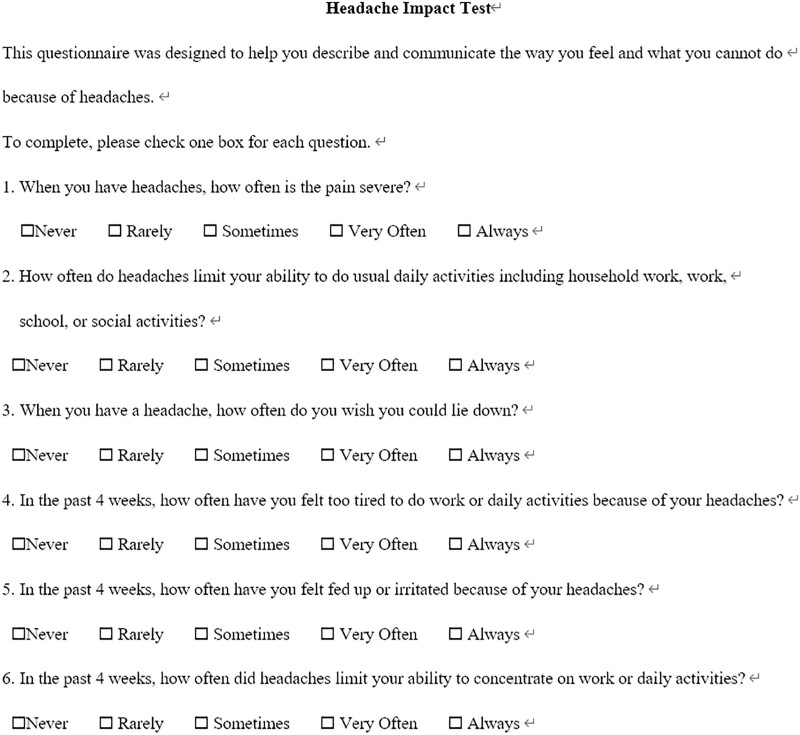
The 6-item headache impact test.

The HIT-6, which has been translated 27 different languages so far,^[15]^ is widely used to assess the impact of headache.^[16]^ It consists of 6 items to assess the intensity of headache and each item is scored using 5 response categories (never, rarely, sometimes, very often, or always), each category of which is associated with a value (6, 8, 10, 11, and 13 respectively), resulting in a range of possible total summed scores of 36 to 78 (Fig. 2).

According to the Delphi study by Luedtke, HIT-6 and headache frequency are the most useful and meaningful outcome for research on the effectiveness of non-pharmacological intervention for headache and migraine.^[17]^ The frequency of headaches is determined based on the patient daily headache diary or direct feedback from the subject.

#### 2.6.2. Secondary outcome: The scores of HDI and the HAMA; the safety and feasibility.

##### The Henry Ford Headache Disability Inventory (HDI):

**Figure 3.**
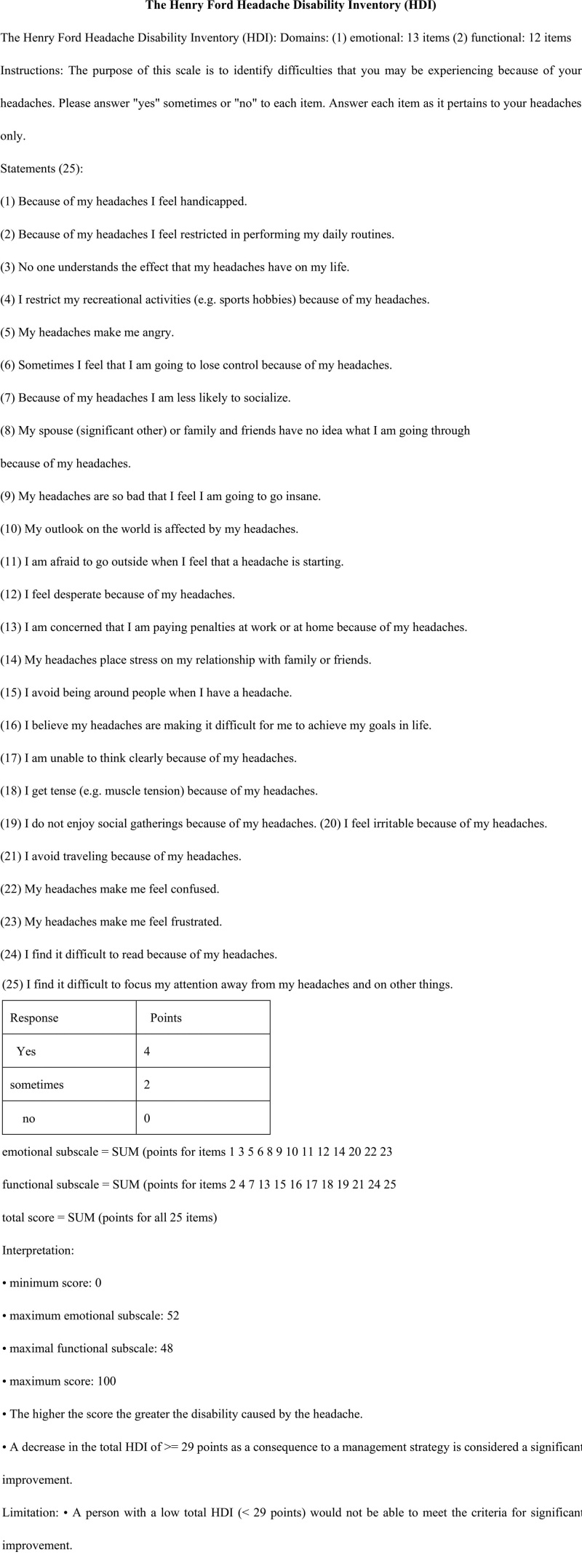
The Henry Ford Headache Disability Inventory (HDI).

HDI can be used to periodically evaluate a patient with headache and can be used to determine the effectiveness of a management strategy over time. It is a 40-item self-assessment scale designed to facilitate the clinician assessment of the patient perception of the functional and emotional aspects of their headaches (Fig. 3). HDI is a useful measure for quantifying the impact of headache on daily living and it has been shown to have good internal consistency, construct validity, and test-retest reliability and has been used to measure the impact of headache programs on patient disability.^[18–21]^

##### Hamilton Anxiety Scale (HAMA):

Studies have found that anxiety, especially general anxiety, is very common in migraine patients.^[22]^ This psychiatric comorbidities can promote the transformation of episodic headache into a chronic syndrome^[23,24]^and this transformation may increase the difficulty in both treatment and headache related disabilities.^[25]^ However, the identification and diagnosis of mental comorbidity in migraine patients is very low, especially in China. This may due to the inherent differences from race and culture because Chinese culture tends to deny psychological symptoms, especially depression. Some studies in China detect that the incidence of depression in migraine patients was lower than the rates observed in other clinical studies,^[26,27]^ including 39.3% seen in Spanish^[28]^ and 23.1 % seen in Italy,^[29]^ and are also lower than the rate in a population-based study which ranged from 24.4 to 49.2 % in USA.^[30]^ And anxiety was more robustly associated with increase in migraine risk than depression 2017.^[30]^ Therefore, we just used the HAMA to evaluate the mental complications of migraine patients rather than select the depression score

##### The safety and feasibility:

Safety assessment was obtained by direct contact with research staff or by asking patients about the frequency and the severity of side effect before and after each treatment round. Besides, patients were also required to record side effects and simultaneous treatment and medicine use in daily records. No serious adverse events were reported.

### 2.7. Statistical analyses

The SPSS 17.0 for Windows was used to perform statistical analyses. All values were expressed as the mean ± standard deviation. The data of 2 groups were analyzed using the repeated measurement analysis of variance. Differences are considered statistically significant when *P* < .05.

## 3. Results

**Figure 4.**
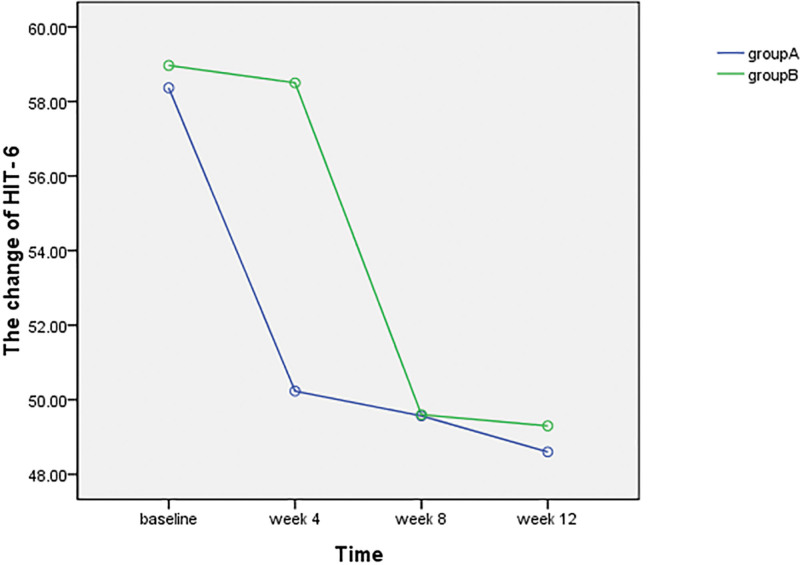
Our rehabilitation center and professional therapist.

All 87 individuals had been screened for eligibility, of which 60 were licensed for the study. Evenly divided into group A and group B. Figure 1 displays the Consort flow chart of patient recruitment and loss during the study period. Out of 87 initial patients, 27 had to be excluded because of not fulfilling the eligibility criteria or other reasons (including secondary migraine, contraindications, comorbidities). In total, 60 patients were randomized and equally allocated to 1 of the 2 treatment groups (group A or group B). Throughout the entire study period, no participants experienced any related injuries or withdrew from the study for any other reason, so no subjects were lost. Figure 4 displays our rehabilitation center and professional therapists. Our rehabilitation center has completed multiple clinical training sessions of CST and treated many sleep disorder and migraine patients, including teenagers, students, and so on. Everyone reported good clinical results.

### 3.1. Baseline data

The age of the subjects ranged from 20 to 50 years old and the average age of group A was 40.7 ± 9.6 years. 12 cases were male; The average age of group B was 38 ± 10.3 years. 11 cases were male. The commonly related factors of migraine were also shown in Table 2. There were no significant differences between 2 groups.

**Table 2:** Baseline characteristics.

Characteristics	Group A	Group B	*P* value
Age (yr)	40.7 ± 9.6	38.2 ± 10.3	.35
Gender (male/female)	12/48	11/49	.79
Systolic pressure (mm Hg)	125.2 ± 12.1	127.1 ± 11.3	.47
Diastolic pressure (mm Hg)	71.2 ± 8.3	75.6 ± 10.1	.06
Insomnia (n)	12	14	.60
Hamilton Anxiety Scale (score)	17.00 ± 3.70	16.83 ± 4.04	.52
Have family history (n)	4	5	.71

Date is presented as mean ± standard (SD); Number (n).

### 3.2. Primary outcome: HIT-6 and Headache frequency

#### 3.2.1. HIT-6.

**Figure 5.**
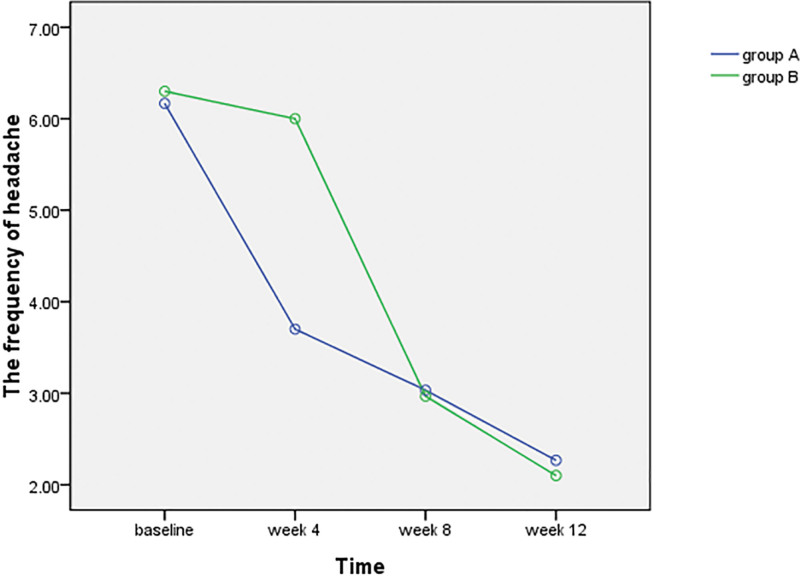
This figure visually shows the change trend of HIT-6 with over time. The score of group A decreased significant from baseline to week 4 and group B decreased obvious from week 4 to week 8.

The HIT-6 are presented in Table 3 and Figure 5. There was no significant difference between the 2 groups at the baseline (*P*^1^ = 0.77 > 0.05, 95% CI, −4.68 to 3.49). After the first round of treatment (group A received CST while group B receive CLST), the score of group A was significantly decreased (*P*^1A^ = 0.01 < 0.05, 95% CI, 4.55–11.7), while group B did not change obviously (*P*^1B^ = 0.79 > 0.05, 95% CI, −3.14 to 4.08). And the difference between the 2 groups at week 4 was obvious (*P*^2^ = 0.01 < 0.05, 95% CI, −11.23 to −5.25). After the second round of treatment (group A and group B interchanged), the score of group A did not change significantly (*P*^2A^ = 0.58 > 0.05, 95% CI −3.09 to 1.76) while the score of group B decreased obviously (*P*^2B^ = 0.01 < 0.05, 95% CI, −11.78 to −6.01). And the difference between the 2 groups was similar (*P*^3^ = 0.97 > 0.05, 95% CI, −11.23 to −5.25). The scores of fourth evaluation showed that there was no significant increase or decrease in 2 groups because both of the groups received no treatment. We also found that the mean scores of HIT-6 for all participants, compared with the baseline, were decreased significantly after the 3 round treatment (*P*^3A^ = 0.01 < 0.05, 95% CI, −13.12 to −6.4; *P*^3B^ = 0.01 < 0.05, CI, −12.73 to −6.69).

**Table 3:** HIT-6 of the 2 groups of patients before and after treatment.

Group	n	Baseline	Wk 4	Wk 8	Wk 12
A	30	58.36 ± 8.47	50.23 ± 4.88^1A^	50.47 ± 5.24^2A^	48.46 ± 4.78^3A^
B	30	58.96 ± 7.32	58.50 ± 6.642^1B^	49.60 ± 4.26^2B^	49.30 ± 4.46^3B^
*P*		.77^1^	.01^2^	.97^3^	.49^4^

Table 3 presents the mean and standard deviation of the total score for the HIT-6 for every group at each time point. There was no difference in HIT-6 between the 2 groups at baseline. Compare with the CLST, the HIT-6 of the groups were significantly decreased after the CST at the week 4 (group A) and week 8 (group B). While the change was not obvious after CLST in group A at week 8 and group B at week 4 compared with prior to treatment.

#### 3.2.2. Headache frequency.

**Figure 6.**
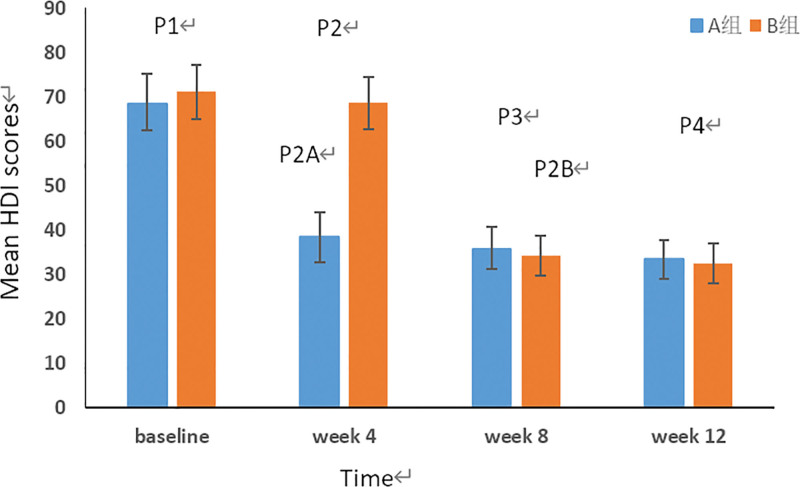
This figure visually shows the change trend of headache frequency with over time. The change of headache frequency in group A from baseline to week 4 and the change of group B from week 4 to week 8 were significant. On the contrary, the change of group A from week 4 to week 8 and the change of group B from baseline to week 8 were not obvious. It indicates that the headache frequency of both groups was significantly reduced after the CST in 2 groups while not obvious after CLST. CLST = complementary light-touch sham treatments. CST = Craniosacral therapy.

According to the criteria for inclusion, the headache frequency of subject was from 5 to 9 per month. The frequency and trend of headache are shown in Table 4 and Figure 6. Table 4 shows the 2 groups had a similar frequency of headache at baseline (P^*^=0.71 > 0.05, 95% CI, −0.84 to 0.57). Group A received CST in the first round of treatment and group B received CST in the second round. Therefore, the headache frequency of 2 groups were all declined respectively after the CST at week 4 (group A) and week 8 (group B) than before and the difference was statistically significant (*P*^☆^=0.01 < 0.05, 95% CI, −3.06 to −1.87; *P*^※^=0.01 < 0.05, 95% CI, −3.52 to −2.53). But there was no significant difference between the 2 groups at week 8 (*P*^□^=0.74 > 0.05, 95% CI, −0.34 to 0.47) and week 12 (*P*^△^=0.44 > 0.05, 95% CI, −0.26 to 0.59) indicating that the effect immediately after CST and 1 month after CST is equivalent.

**Table 4:** Headache frequency changes.

Group	n	Baseline	Wk 4	Wk 8	Wk 12
A	30	6.16 ± 1.28	3.7 ± 0.98^☆^	3.03 ± 0.68	2.26 ± 0.82
B	30	6.30 ± 1.46	6.0 ± 1.07	2.96 ± 0.89^※^	2.11 ± 0.78
*P*		.71^*^	.01^#^	.74^□^	.44^Δ^

Table 4 present the Mean and standard deviation of headache frequency at baseline, after the first round of treatment (week 4) and the second round of treatment (week 8) and 4 wk follow-up observation (week 12) (P^*^=0.71 > 0.05, 95% CI, −0.84–0.57; *P*^☆^=0.01 < 0.05, 95% CI, −3.06 to −1.87; *P*^※^=0.01 < 0.05, 95% CI, −3.52 to −2.53; *P*^□^=0.74 > 0.05, 95% CI, −0.34 to 0.47; *P*^Δ^=0.44 > 0.05, 95% CI, −0.26 to 0.59).

### 3.3. Secondary outcome: The scores of HDI and the HAMA

#### 3.3.1. The change of HDI scores.

**Figure 7.**
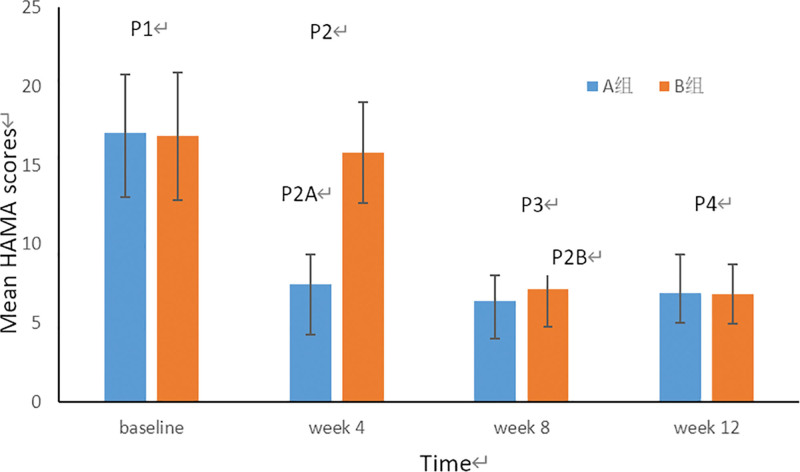
The change of HDI scores at the different treatment time points. The blue histogram represents the HDI scores of group A before and after every round treatment and the red histogram represents the HDI scores of group B. The value of *P*^1^, *P*^2^, *P*^3^, and *P*^4^ represents the comparison between the 2 group at different treatment time points respectively (*P*^1^ > 0.05, *P*^3^ > 0.05, *P*^4^ > 0.05). The value of *P*^2A^ and *P*^2B^ represents the comparison between the same group before and after CST at week 4 and week 8 respectively. (*P*^2A^ < 0.05, *P*^2B^ < 0.05). CST = Craniosacral therapy, HDI = Headache Disability inventory.

The average scores of HDI in migraine patients were analyzed at the different treatment time points respectively. As we can see in Figure 7, the average scores of 2 groups was similar at baseline (*P*^1^ = 0.12 > 0.05, 95% CI, −5.95 to 0.75). After group A received CST in the first round and group B received CST in the second round, the scores of 2 groups were all declined respectively than before and the difference was statistically significant (*P*^2A^ = 0.01 < 0.05, 95% CI, 26.79–33.2; *P*^2B^ = 0.01 < 0.05, 95% CI,31.72–37.07). But there was no significant difference between the 2 groups at week 8 (*P*^3^ = 0.17 > 0.05, 95% CI, −0.78 to 4.11) and week 12 (*P*^4^ = 0.37 > 0.05, 95% CI, −0.3.25 to 1.25).

#### 3.3.2. The change of HAMA scores.

**Figure 8.**
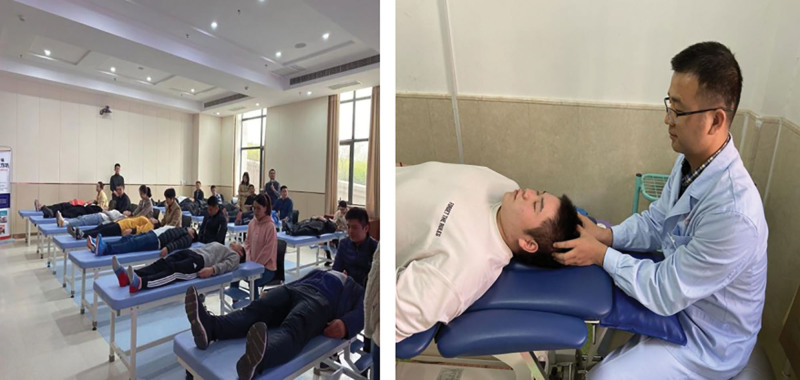
The change of HAMA scores at the different treatment time points. The blue histogram represents the HAMA scores of group A before and after every round treatment and the red histogram represents the HAMA scores of group B. The value of *P*^1^, *P*^2^, *P*^3^, and *P*^4^ represents the comparison between the 2 group at different treatment time points respectively (*P*^1^ > 0.05, *P*^3^ > 0.05, *P*^4^ > 0.05). The value of *P*^2A^ and *P*^2B^ represents the comparison between the same group before and after CST at week 4 and week 8 respectively. (*P*^2A^ < 0.05, *P*^2B^ < 0.05). CST = Craniosacral therapy, HAMA = Hamilton Anxiety Scale.

The incidence of anxiety symptoms was 68.3 % (41of 60) in our samples (HAMA > 7) which was similar to the results of a study from Spanish (69 %).^[28]^ As shown in Figure 8, the 2 groups had similar average scores at baseline, week 8 and week 12 (*P*^1^ = 0.16 > 0.05, 95% CI, −1.83 to 2.17; *P*^3^ = 0.45 > 0.05, 95% CI, −1.46 to 0.66; *P*^4^ = 0.06 > 0.05, 95% CI, −1.18 to 1.05;). But the difference was statistically significant between and within 2 groups after CST treatment (*P*^2^ = 0.01 < 0.05, 95% CI, −9.68 to −6.98; *P*^2A^ = 0.01 < 0.05, 95% CI,8.01–11.04; *P*^2B^ = 0.01 < 0.05, 95% CI, −10.11 to −7.21). we can also find that the trend of anxiety score is consistent with HT-6 and migraine attack frequency.

## 4. Discussion

In the 1930s, an osteopath named William Sutherland laid the groundwork for CST, after working extensively with patients who experienced a wide range of symptoms^.[31]^ It is one popular no-pharmacological approach to the treatment or prevention of neck pain, migraine headaches and neuromuscular dysfunction. It is in widespread use in both the United Kingdom and United States. But fewer researches on Asian populations have been reported. As one non-pharmacological and alternative interventions, CST has been being researched for recent years in China. At present, “Craniosacral Therapy Course,” a professional book finished by Rongke Di, had been published by Jiangsu University Press on December, 2017 in China. The craniosacral system is defined as a recognized, functioning physiological system, including the membranes and cerebrospinal fluid surrounding the spinal cord and brain, the bones to which these membranes attach and connective tissue related to these membranes.^[32]^ It is intimately related to and influenced by the nervous, musculoskeletal, lymphatic, vascular, endocrine and respiratory system of the body.^[32]^ The craniosacral system is characterized by rhythmic and mobile activities, but it is completely different from the physiological movements related to breathing and cardiovascular activities.^[32]^

CST is based on the theory that the movement restriction at the cranial sutures of the skull negatively affects the rhythmic impulses transmitted from the skull to the sacrum through the cerebrospinal fluid.^[33]^ All structures in contact with the cerebrospinal fluid, including the brain, spinal cord and its protective membrane, are considered as part of the craniosacral system and may be affected by it. All other structures in the body are potentially affected indirectly through innervations originating from the central nervous system or returning to the central nervous system, or directly through the activity of the musculoskeletal system. Therefore, the purpose of craniosacral treatment is to remove the restrictions around the spinal cord and brain, and then restore body function.

While the specific mechanisms of CST are still understudied, clinical trials have shown preliminary evidence for CST on improving patient-reported outcomes.

Upledger believes that the sutural immobility of the skull is a contributing factor to migraine.^[13]^ And therapists in this research found these patients experienced migraine pain when the technique for the sutures was performed.^[13]^ In addition, this study also believes that there is a correlation between the disorder of craniosacral rhythm and migraine, which is closely related to the contraction of the ventricle, the cranial endometrial system and expansion. When the body is in a pathological state, the “cranial sacral rhythm” will be abnormal. Through using gentle intensity to touch the Cranial sacrum and the patient craniosacral system, the rhythm and flow of cerebrospinal fluid will be adjusted to normal by the therapist. This will directly regulate the functional status of the brain and spinal cord, restore the normal connection and natural movement of the central nervous system and other body systems, adjust the balance of the body various systems, remove the body metabolites, and enhance the body self-healing function. So, it is widely used in different environments and conditions of diagnosis and treatment.

Our center is a rehabilitation institution that develop and research craniosacral treatment and has our own professional therapists and treatment rooms for many years (Fig. 5). This article was based on a prospective study of Asian populations with the purpose of investigation on the effects of CST for migraine headaches. In order to reduce sample bias and improve objectivity, participants in this study were all indoor white-collar workers who had received higher education, and a cross-controlled trial where the 2 groups functioned as their own comparison group was used. Our experimental results demonstrate that the scores of the HIT-6 Questionnaire and the frequency of migraine were significant reduced for both groups at point immediately after every cycle of CST treatments. As is known anxiety and mood disorders have been shown to be the most relevant psychiatric comorbidities associated with migraine, influencing its clinical course, treatment response, and clinical outcomes. Comparison across the various headache non-pharmacological groups showed that headache related disability, as measured by the HDI, decreased as mean Headache Impact Test for patient groups decreased. And so as the trend of anxiety score and migraine attack frequency. This result was similar to the result of a study from Jane E.^[18]^ But the incidence of anxiety symptoms was 68.3 % in our samples, which was higher than the rates observed in other clinical studies in other western countries,^[29,34]^ except the result from Spanish (69 %).^[28]^ These differences may due to economic and cultural differences or different evaluation instruments. For example, the life pattern in mainland China may be different than that in Western countries or Taiwan. On the other hand, we choose Hamilton anxiety score > 7 as the diagnostic criteria which lowers the inclusion criteria. The third reason may be the participants in this study were all indoor mental worker who had received higher education. Their work disposition and environment may lead to a high incidence of anxiety symptoms.

All the 60 enrolled subjects completed the treatment without any fall-off, and no obvious adverse reaction report was received at the end of the treatment. The results indicated that standardized CST was both effective and safe in alleviating the migraine intensity and frequency as well as the Headache-related disability. This study provides new evidence on the effect of CST on migraine in Asians. This is important because migraine is a common disease that seriously affects people health, especially for those indoor white-collar workers.

## 5. Limitations

The limitations of this study are the small sample size, and the potential differences in skills and techniques of therapists, even though we participated in 3 therapists. On the other hand, this is a single center clinical study and participants in this study were all indoor white-collar workers who had received higher education. The singularity of the subject profession and work environment is also a limitation of this study. It would be interesting to repeat this study with additional measurement tools, and it is also recommended to conduct large-scale multi-center clinical research and extend the follow-up time and the long-term effects of CST. Although this study may provide valuable results to further support clinical decision-making, further research is needed.

## Acknowledgments

The authors are grateful to all staff members, doctors, and all participants who were involved in this study.

## Author contributions

**Conceptualization:** JinFeng Zhao, Lin Cui, LiuYun Jia.

**Data curation:** SaiChao Ma, LiuYun Jia.

**Funding acquisition:** LiuYun Jia.

**Formal analysis:** SaiChao Ma, Yan Li.

**Investigation:** JinFeng Zhao, Yan Li.

**Methodology:** GuangYa Jiang, JinFeng Zhao.

**Project administration:** Wenli Chen, Lin Cui.

**Resources:** Yan Li.

**Supervision:** Wenli Chen.

**Validation:** Ming Zhang.

**Visualization:** Ming Zhang, Wenli Chen, LiuYun Jia.

**Writing – original draft:** GuangYa Jiang.

**Writing – review & editing:** Ming Zhang, Wenli Chen, LiuYun Jia.
